# Cross-cultural validity of four quality of life scales in persons with spinal cord injury

**DOI:** 10.1186/1477-7525-8-94

**Published:** 2010-09-03

**Authors:** Szilvia Geyh, Bernd AG Fellinghauer, Inge Kirchberger, Marcel WM Post

**Affiliations:** 1 Swiss Paraplegic Research (SPF), Nottwil, Switzerland; 2 Department of Health Sciences and Health Policy, University of Lucerne and SPF, Nottwil, Switzerland; 3 Seminar for Statistics, Swiss Federal Institute of Technology, Zurich, Switzerland; 4 Institute of Health and Rehabilitation Sciences, Ludwig Maximilian University, Munich, Germany; 5 Rehabilitation Centre 'De Hoogstraat' and Rudolf Magnus Institute for Neuroscience, Utrecht, The Netherlands

## Abstract

**Background:**

Quality of life (QoL) in persons with spinal cord injury (SCI) has been found to differ across countries. However, comparability of measurement results between countries depends on the cross-cultural validity of the applied instruments. The study examined the metric quality and cross-cultural validity of the Satisfaction with Life Scale (SWLS), the Life Satisfaction Questionnaire (LISAT-9), the Personal Well-Being Index (PWI) and the 5-item World Health Organization Quality of Life Assessment (WHOQoL-5) across six countries in a sample of persons with spinal cord injury (SCI).

**Methods:**

A cross-sectional multi-centre study was conducted and the data of 243 out-patients with SCI from study centers in Australia, Brazil, Canada, Israel, South Africa, and the United States were analyzed using Rasch-based methods.

**Results:**

The analyses showed high reliability for all 4 instruments (person reliability index .78-.92). Unidimensionality of measurement was supported for the WHOQoL-5 (Chi^2 ^= 16.43, df = 10, p = .088), partially supported for the PWI (Chi^2 ^= 15.62, df = 16, p = .480), but rejected for the LISAT-9 (Chi^2 ^= 50.60, df = 18, p = .000) and the SWLS (Chi^2 ^= 78.54, df = 10, p = .000) based on overall and item-wise Chi^2 ^tests, principal components analyses and independent t-tests. The response scales showed the expected ordering for the WHOQoL-5 and the PWI, but not for the other two instruments. Using differential item functioning (DIF) analyses potential cross-country bias was found in two items of the SWLS and the WHOQoL-5, three items of the LISAT-9 and four items of the PWI. However, applying Rasch-based statistical methods, especially subtest analyses, it was possible to identify optimal strategies to enhance the metric properties and the cross-country equivalence of the instruments post-hoc. Following the post-hoc procedures the WHOQOL-5 and the PWI worked in a consistent and expected way in all countries.

**Conclusions:**

QoL assessment using the summary scores of the WHOQOL-5 and the PWI appeared cross-culturally valid in persons with SCI. In contrast, summary scores of the LISAT-9 and the SWLS have to be interpreted with caution. The findings of the current study can be especially helpful to select instruments for international research projects in SCI.

## Background

In the general population, quality of life (QoL) is measured across countries to indicate the state and development of societies like, for example, in the annual Eurobarometer of the European Commission [1] or the World Values Survey [2]. National levels of QoL have been found to be related with wealth, human rights, individualism, and the fulfillment of basic biological needs in a given society [3,4]. Measuring QoL of individuals with certain health conditions provides information about health states beyond diagnosis, about the impact of a disease and its treatment on different domains of daily life, and about the health experience from the "insider" perspective of the affected persons themselves [5,6]. In relation to health, QoL is measured across countries to compare the burden of disease and disability in different populations. However, QoL is not restricted to health-related issues.

The notion of QoL in general covers various concepts including health-related quality of life (HRQoL) but also subjective well-being (SWB) [7]. HRQoL, on the one hand, describes difficulties caused by poor health on mental and physical functioning, task performance, participation in life areas, or "health status" [8,9]. SWB on the other hand, includes overall life satisfaction, satisfaction with life domains, as well as positive and negative affect [10]. Life satisfaction is traditionally viewed as a cognitive, needs-based approach towards QoL. It refers to the individual's personal evaluation of the gap between his or her aspirations and achievements. More currently, also a cognitive-affective conceptualization of satisfaction has been discussed [10,11].

Essentially, life satisfaction is related to the subjective "insider" perspective and is increasingly considered as a meaningful and efficient way to collect information about QoL [12,13]. Assessing QoL of individuals in health services provision and research complements measurement that is based on performance, and adds relevant information for treatment decision-making and outcome evaluation [6,14].

QoL of persons who sustained spinal cord injury (SCI) seems to be diminished compared to the general population [15,16] QoL appears not to be directly related to the severity of SCI [16,17], but it is related to perceived health, participation and integration, to social support and relationships as well as to living circumstances, e.g. accessibility or income [15,17].

Several reviews summarized the application and metric properties of QoL measures in SCI [16,18-20]. Among the various instruments with promising properties were also short scales, such as the Satisfaction with Life Scale (SWLS) [21], which is part of the United States SCI Model Systems [22], the Life Satisfaction Questionnaire (LISAT) [23], or the World Health Organization Quality of Life Assessment (WHOQOL-BREF) [24].

QoL in persons with SCI has been found to differ across countries [25,26]. Such differences may be related to country level factors (e.g. culture and values), to internal and external individual level factors (e.g. personality, self-esteem or social support), as well as their interactions (e.g. social desirability) [27]. Differences found in these studies may reflect the properties of the measurement instruments used.

The comparability of measurement results between countries depends on the cross-cultural validity of the applied instruments [28]. Common steps in various guidelines for cross-cultural adaptation of QoL instrument include systematic translation procedures and cross-cultural testing of psychometric properties [29]. There have been efforts to develop and/or validate QoL instruments cross-culturally (e.g. the WHOQoL-development or the International Quality of Life Assessment project) [30,31]. However, the cross-cultural validity and international comparability of QoL measurement is not well established in SCI.

Psychometric properties, like reliability, validity, etc. can be examined using different techniques. Currently, Rasch-based methods are becoming increasingly popular in the context of rehabilitation outcome measurement [32]. They are used to create interval scale measurement, can reveal metric difficulties of the measures, but also provide techniques to account for them at a statistical level in certain circumstances, for example, by item reduction, collapsing response scale options, splitting items, etc. Thus, Rasch-based methods have also been used to examine and account for cross-cultural bias in outcome measures [33,34].

The objective of this study is to examine the cross-cultural validity of selected QoL scales across countries in a sample of persons with SCI using Rasch analysis. The specific aims are (1) to examine and compare measurement properties of the instruments, namely, dimensionality, response scale structure, and reliability; (2) to examine the validity of the instruments across countries; and (3) to examine possibilities to enhance the measurement properties and the cross-cultural validity of the instruments.

## Methods

### Design and setting

This cross-sectional multi-centre study was conducted as a nested project within the international collaborative development of the "ICF Core Sets for Spinal Cord Injury" [35,36]. For the current analyses, data from participating study centers in Australia, Brazil, Canada, Israel, South Africa, and the United States are used.

### Participants and data collection

Subjects were recruited through the six participating rehabilitation facilities. Patients were recruited who had sustained a SCI with an acute onset and who were at least 18 years old. Acute onset was defined as a trauma or non-traumatic event resulting in spinal cord dysfunction within 14 days of onset. Subjects with significant traumatic brain injury or diagnosed mental disorders prior to SCI were excluded. Prior to data collection participants were informed about the purpose and reason of the study and signed an informed consent.

For the purpose of the analyses presented in this paper data from outpatients were selected. In four of the participating centers data were also collected for inpatients. Overall, 109 inpatient data sets were available; however, 76% of these were from one country only (Israel). Thus, to avoid confounding of country with care setting, and to obtain a more homogeneous data set for the cross-country comparisons, the inpatient data were omitted.

The data collection included, beside socio-demographic and injury related variables, four QoL measures: The Satisfaction with Life Scale (SWLS) [21], the Life Satisfaction Questionnaire-9 (LISAT-9) [23], the Personal Well-Being Index (PWI) [37] and five satisfaction items from the World Health Organization Quality of Life Assessment (WHOQOL-5) [24,38]. For the data collection, instruments were selected that include less than 10 items, focus on the concepts of life and domain satisfaction, and contain items that are applicable and not offensive to people with SCI (do not contain items on walking, kneeling, bending, etc.). In addition, psychometric properties and the availability of different language versions were considered. Short questionnaires are more feasible, acceptable, and impose less burden on the patients compared to longer instruments. They can be more easily embedded into routine clinical assessments or larger scale data collection schemes. Instruments were chosen with a focus on the aspect of satisfaction within the broader notion of QoL, as satisfaction is not only conceptually well-defined, but has also been traditionally considered as a clinically relevant person-centered outcome in rehabilitation [39].

In Australia, Canada, South Africa, and the United States the English versions of the instruments were used. For the SWLS and the WHOQOL also the Portuguese (Brazil) and the Hebrew (Israel) versions exist. However, for the LISAT and the PWI translations were not available in Brazil and Israel. In these cases, translations of the English version were prepared at the participating facilities.

### Satisfaction with Life Scale

The SWLS was designed to assess global life satisfaction. It addresses the cognitive evaluation of one's own life in terms of ideal life, wish for change, and current and past satisfaction. The SWLS consists of five items with a 7-point Likert-scale from "strongly disagree" to "strongly agree". Reliability and validity of the scale have been examined in several studies [21,40,41] also for various translations and in different countries [42,43]. The SWLS has been used in cross-country studies in the general and student populations [27] and is also widely used in SCI research, especially in the United States [22,44-49]. Internal consistency coefficients range between .79 and .89 [40] and several studies confirmed the single factor structure of the SWLS [21,41-43,50]. However, studies in SCI scarcely reported about the psychometric properties of the instrument [47]. Two studies comparing general population samples in the United States and Russia [51], Norway and Greenland [52], respectively, hinted at potential cross-cultural biases affecting the interpretation of the SWLS.

### Life Satisfaction Questionnaire

The LISAT-9 is a measure of domain-specific life satisfaction. It consists of nine items including one on general life satisfaction and eight domain-specific items (self-care, vocational, financial, leisure situation, sexual life, partner relationship, family life, social contacts). Responses are rated along a 6-point scale from "very dissatisfying" to "very satisfying". Among the psychometric properties of the LISAT, internal consistency and factorial structure are reported in the literature [23,53,54]. A 3-factor has been shown for the LISAT-9 and a 4-factor structure for the LISAT-11 with internal consistency reliability of the factors between .57 and .79 (overall .85) [23,53]. Thus, analyses using the LISAT are frequently done item-wise, but also using mean or median of the scores. The instrument has been widely used in SCI research, mainly in Europe [25,54-59], little is known about the measurement properties of the LISAT in non-European countries, and only few studies have addressed the psychometric properties of the LISAT in the SCI population [54] The LISAT has also been used to compare SCI samples across countries (Sweden and Japan; China and UK; UK, Germany, Austria, and Switzerland), however, without considering potential cross-cultural validity issues [25,26,58].

### Personal Well-Being Index

The PWI consists of 7 items about satisfaction with specific life domains (living standard, health, achievement, relationships, safety, community, future security) and one optional item about overall life satisfaction. Responses are provided on a 0-10 numeric rating scale with the end points "completely dissatisfied" to "completely satisfied". The PWI has been developed in Australia for use in national surveys [60] and has been adapted for international use [37]. Validity and reliability of the PWI have been demonstrated in general population samples from different countries [37,60-62]. The PWI has been designed as a unidimensional tool with internal consistencies between .70 and .85. Although already used in various countries (Australia, Hong Kong/China, Algeria), a rigorous examination of cross-cultural validity has not yet been conducted. The PWI has not been used with persons with SCI so far.

### World Health Organization Quality of Life Assessment-5

The WHOQOL-5 is a selection of five satisfaction items out of the World Health Organization's short health-related quality of life measure, the WHOQOL-BREF. The 5 items cover overall quality of life, satisfaction with health, daily activities, relationships, and living conditions. The WHOQOL and WHOQOL-BREF were specifically developed for cross-cultural use and are currently available in 36 languages. Psychometric properties have been examined in 23 countries with samples of sick and healthy persons [24,38,63], with internal consistency coefficients lying between .75 and .87. The WHOQOL-BREF has also been applied in people with SCI [64,65]. A selection of 8 items out of the WHOQOL-BREF (including the 5 items in this study) was used in the EUROHIS project across 10 European countries and showed satisfactory psychometric properties, unidimensionality and cross-cultural validity [66,67]. The 5-item version has been used in different international WHO collaboration projects since 2002 [35,68,69], but has not been psychometrically tested previously in this format.

### Ethics committee approval

The study was carried out in compliance with the Helsinki Declaration, the design and materials were approved by the Ethics Committee of the Ludwig-Maximilian University Munich, as well as by the respective Ethics Committees for the study centers in each world region.

### Rasch Analyses

Rasch analyses were carried out using the RUMM software [70] and applying the partial credit Rasch model [71]. This model is a special case of the one-parameter Rasch model. In the field of Rasch-based or item response modeling further types of models exist, e.g. two- or three-parameter item response models, nonparametric Mokken analyses, or mixed Rasch models, etc. The use of these models might result in better fit of the data, as they consider varying item difficulty curves, varying homogeneity or monotonicity of the data, or multiple latent classes within the sample populations. However, the one-parameter Rasch model is especially helpful for developing precise and accurate measurement instruments, as it imposes strict requirements on the items and is not data-driven. It can ensure through its mathematical formulation fundamental measurement in the tradition of Guttman's work within a probabilistic framework [72,73].

Applying this type of Rasch analysis, three parameters are estimated: The person parameters (for the patients), the item parameters, and the parameters of the thresholds of the response scale (e.g. four threshold parameters for a 5-point Likert-scale). These parameters describe the position of the persons, items and thresholds on the unidimensional continuum of the measured latent trait (e.g., low to high quality of life).

First, the unidimensionality of each instrument was examined. Unidimensionality describes the idea that items should contribute to the measurement of only one attribute at a time and should not be confounded by other attributes or dimensions [73]. This ensures the interpretability of the summary scores of the instrument. Unidimensionality can be checked for by comparing the observed responses in a set of items to the expected values predicted by the unidimensional Rasch model [74]. The fit of each item is indicated by standardized residuals (z values) and Chi^2 ^test results. Z values exceeding +/-2.5 are considered to indicate misfit to the Rasch model [74]. For the Chi^2 ^significance tests a Bonferroni-corrected critical p-value at the 5% level [75] was applied.

To further examine unidimensionality, principal components analyses (PCA) of the residuals not explained by the Rasch-model were performed. The residuals should show a random pattern to indicate unidimensionality [76]. Given the sample size in this study, eigenvalues below 1.9 in the PCA results are indicative of random residual variation, eigenvalues above 1.9 indicate some structure in the residuals [77]. In addition, the Rasch person parameters of each patient were estimated separately for the items with positive versus negative loadings on the first PCA factor, and then compared using independent t-tests. The percentage of significant t-tests (α = 0.05) should not exceed 5% [78,79].

The structure of the response scale for each instrument was studied based on the ordering of the threshold parameters. The threshold parameters should take increasing values, as they represent the successive transition points along the response scale from low to high quality of life. Reversed thresholds show that the scores do not differentiate as intended [80].

Reliability is indicated by the person reliability index, which is the Rasch-based correspondent to Cronbach's alpha [71,81]. The person reliability index is constructed using the person parameter estimates and the standard errors of measurement to calculate the ratio of true person ability variance to the observed variance [74,82]. It ranges between 0 and 1, where the value of 1 indicates perfect reproducibility of person placements on the latent continuum.

To examine the cross-cultural validity of the four instruments across countries, differential item functioning (DIF) analyses were conducted [33]. Potential DIF is ascertained for each item by comparing the standardized residuals between the countries and across the latent trait continuum of QoL using a two-way analysis of variance (ANOVA). A significant main effect of the country (uniform DIF) or a significant interaction effect in the ANOVA results (e.g. Country × QoL, non-uniform DIF) indicates problems with the cross-country comparability of the responses. If no DIF is apparent, the scores are comparable across countries. A respective Bonferroni-corrected type I error level was applied [75]. Tukey-Cramer post-hoc tests allowed identifying the countries that contribute to DIF in the data.

**Figure 1:**
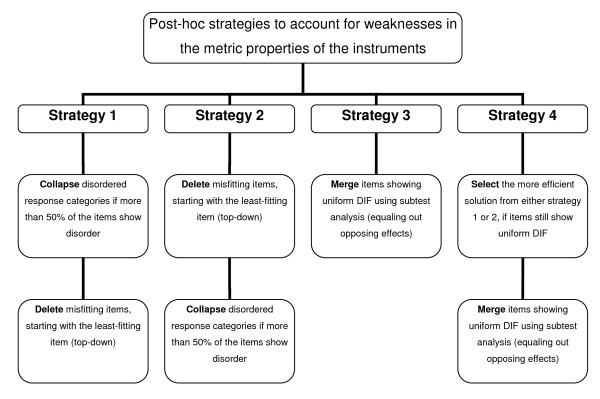
**Overview of the four Rasch-based strategies applied to account for the weaknesses in the metric properties of the four quality of life instruments post-hoc**.

Based on the results of Rasch analyses different approaches can be taken to account for weaknesses in the metric properties of the instruments post-hoc. To come up with suggestions to enhance the measurement properties and cross-cultural validity of the instruments across countries, four alternative strategies of handling the data set were tested and compared. As a result, for each instrument an optimal solution for handling the data could be identified, which allows for acceptable measurement properties with as little change to the instrument as possible. Figure 1 gives an overview of the four strategies implemented in the post-hoc analyses.

In the first strategy, response scale disorder was addressed first. Disordered response categories were collapsed, i.e. adjacent response options were merged and the scores recoded for all items of the instrument if more than half of the items showed disorder [80]. In addition, items that still misfitted after the collapsing, were deleted using a step-wise top-down deletion strategy until the remaining items fit the model [83].

In the second strategy, item misfit was attended to first by using the step-wise top-down deletion strategy and the remaining fitting items are checked again for response scale disorder.

The third strategy focused on accounting for DIF. So-called subtest analyses were conducted, which were used to merge the scores of those items that display DIF for country. Thereby, if two items of an instrument show DIF but in opposite directions, they can be combined into one score, which adjusts for invariance across countries. The advantage of this strategy-if it is successful in ameliorating DIF-is that no changes to the items are necessary and the summary score of the instrument can be interpreted as comparable across countries.

The fourth strategy also addressed DIF, but applied the subtest analyses to either option one or option two, depending on which of the two represented the most effective strategy for the instrument so far (i.e. enhanced statistics with less change).

The strategies one to three were calculated for all four instruments (according to the properties in the basic analyses), and after each step, the overall and item fit, DIF, response scale ordering, and reliability were documented. The fourth strategy was only applied, if the first three did not result in acceptable metric properties.

The efficiency of the different strategies was determined by the metric properties on the one side and the modifications to the instrument on the other side. Hereby, the metric properties were considered hierarchical in terms of desirability: Item and overall fit were considered the most important criteria to be fulfilled first, DIF as second, and response scale ordering as the third criterion. Regarding the modifications to the instruments, the merging strategy was considered the least invasive strategy, as it does not require changes to the items or the response scale. Collapsing of response options was considered the second least invasive strategy, as it requires the recoding of responses, but no changes to the items. Deletion of items was considered an invasive strategy, as it alters the instrument from its original version.

Thus, if for example the strategies one to three all resulted in acceptable metric properties in terms of fit, DIF, and response scale ordering, then the merging strategy three would be preferred as optimum solution, for being least invasive.

## Results

From six countries and four different world regions, overall, 243 out-patients with SCI were included in the study. Table 1 shows the socio-demographic and SCI-related characteristics of the study sample. Table 2 shows the mean raw scores, respective standard deviations, and the number of valid responses in the four instruments overall, per item, and per country.

**Table 1:** Socio-demographic and spinal cord injury related patient characteristics (N = 243)

Variable	Descriptive statistics
***Socio-demographic characteristics***	
Years of age	
mean (SD)	41.4 (13.6)
	
Gender	
% male	79.4
	
Marital status	
% never married	41.6
% currently married or cohabiting	39.9
% separated, divorced, widowed	18.0
	
Years of education	
mean (SD)	13.0 (4.0)
	
Current occupational situation	
% paid work, self-employed	33.7
% unemployed for health reasons	33.3
% retired	11.5
% other (student, house-maker, etc.)	21.5
	
***Spinal cord injury characteristics***	
Etiology	
% sport	9.1
% assault	6.2
% transport	35.0
% fall	11.9
% other traumatic	20.6
% non-traumatic	7.0
% unspecified	10.3
	
Level of injury	
% paraplegia	45.7
	
Completeness of injury	
% complete (A)	47.7
% incomplete (B-D)	43.6
% unspecified	8.6
	
Time since onset in months	
mean (SD)	139.6 (138.8)
median (IQR)	86.1 (175.1)

**Table 2:** Raw scores for the four instruments overall and by country

Items	ALL			AUS			BRZ			CAN			ISR			RSA			USA		
							
	N	m	sd	n	m	sd	n	m	sd	n	m	sd	n	m	sd	n	m	sd	n	m	sd
***SWLS***																					
*Sum score*	*243*	*18.2*	*7.4*	*40*	*17.2*	*6.0*	*34*	*17.3*	*7.5*	*34*	*20.2*	*7.7*	*71*	*19.3*	*7.1*	*30*	*14.1*	*6.7*	*34*	*19.6*	*8.5*
Ideal life	243	3.3	1.9	40	2.9	1.4	34	3.5	1.8	34	4.1	2.1	71	3.3	1.8	30	2.5	1.7	34	3.6	2.2
Life conditions	243	3.5	1.9	40	3.4	1.5	34	3.7	1.7	34	4.2	2.0	71	3.5	1.9	30	2.7	1.8	34	3.7	2.2
Life satisfaction	243	4.0	1.9	40	4.1	1.4	34	3.7	2.0	34	4.4	2.0	71	3.9	1.9	30	3.6	1.9	34	4.3	2.1
Got things I want	243	3.9	1.8	40	4.0	1.4	34	3.5	2.0	34	4.6	1.7	71	3.8	1.8	30	3.0	1.4	34	4.4	1.8
Change nothing in life	243	3.5	1.9	40	2.9	1.4	34	2.9	1.8	34	3.0	1.7	71	4.8	1.9	30	2.3	1.3	34	3.5	2.0
																					
***LISAT-9***																					
*Sum score*	*243*	*31.6*	*9.4*	*40*	*40.0*	*17.2*	*34*	*31.4*	*9.8*	*34*	*34.8*	*9.9*	*71*	*31.5*	*9.5*	*30*	*27.1*	*7.8*	*34*	*34.1*	*10.3*
Life as a whole	243	3.9	1.3	40	4.0	0.9	34	3.6	1.3	34	4.2	1.3	71	3.9	1.4	30	3.4	1.2	34	4.4	1.2
Self care	243	3.5	1.7	40	2.6	1.4	34	3.5	1.7	34	4.2	1.6	71	3.5	1.6	30	3.1	1.6	34	4.0	1.8
Vocational situation	240	3.4	1.6	40	3.0	1.3	34	3.3	1.5	33	4.1	1.5	70	3.1	1.9	30	3.2	1.2	33	3.8	1.8
Financial situation	243	3.3	1.5	40	2.8	1.1	34	2.9	1.5	34	3.9	1.2	71	3.9	1.6	30	2.5	1.2	34	3.4	1.8
Leisure situation	243	3.4	1.5	40	3.9	0.9	34	2.9	1.3	34	3.8	1.4	71	3.1	1.8	30	3.5	1.3	34	3.6	1.5
Sexual life	237	2.5	1.5	38	2.5	1.4	33	2.5	1.5	32	3.0	1.7	71	2.2	1.6	30	2.2	1.3	33	3.0	1.6
Partner relations	139	4.5	1.6	18	4.7	1.7	25	4.8	1.2	18	4.5	1.7	51	4.0	1.8	8	4.3	1.4	19	5.1	1.1
Family life	242	4.6	1.3	40	5.0	0.8	34	4.7	1.1	33	4.8	1.3	71	4.4	1.5	30	3.7	1.3	34	4.8	1.1
Contact with friends	240	4.6	1.2	38	4.8	0.9	34	4.4	1.2	34	4.7	1.2	71	4.6	1.4	30	4.4	1.1	33	4.6	1.1
																					
***PWI***																					
*Sum score*	*242*	*48.3*	*15.6*	*40*	*43.4*	*10.9*	*34*	*46.9*	*14.9*	*33*	*53.9*	*14.7*	*71*	*47.3*	*17.5*	*30*	*48.9*	*11.3*	*34*	*51.5*	*18.7*
Whole life	242	5.8	2.4	40	5.5	1.8	34	5.8	2.2	33	6.7	2.6	71	5.7	2.6	30	5.1	2.6	34	6.2	2.6
Living standard	242	6.0	2.4	40	5.6	1.9	34	5.3	1.9	33	6.8	2.4	71	6.1	2.5	30	6.1	2.6	34	6.4	2.8
Health	242	5.4	2.6	40	4.3	2.1	34	6.4	2.2	33	5.1	2.9	71	4.9	2.6	30	6.4	2.5	34	6.3	2.4
Life achievement	242	6.1	2.4	40	5.9	1.9	34	5.7	2.3	33	6.5	2.2	71	6.2	2.5	30	6.1	2.3	34	5.8	3.3
Relationships	241	7.0	2.2	40	7.1	1.6	33	7.1	2.3	33	7.1	2.1	71	7.1	2.2	30	6.7	2.2	34	6.6	3.1
Feeling safe	242	6.3	2.7	40	5.1	1.9	34	5.6	2.5	33	7.7	2.4	71	6.1	3.0	30	6.7	2.0	34	7.2	2.8
Feel part of community	242	6.2	2.4	40	6.0	1.3	34	6.1	2.3	33	7.3	1.8	71	5.7	2.8	30	6.0	2.4	34	6.7	2.8
Future security	242	5.5	2.6	40	4.0	2.0	34	5.6	2.3	33	6.7	2.4	71	5.5	2.8	30	5.6	2.3	34	6.3	2.7
																					
***WHOQoL-5***																					
*Sum score*	*243*	*18.2*	*7.4*	*40*	*17.2*	*6.0*	*34*	*17.3*	*7.5*	*34*	*20.2*	*7.7*	*71*	*19.3*	*7.1*	*30*	*14.1*	*6.7*	*34*	*19.6*	*8.5*
Health	243	3.3	1.0	40	3.1	0.9	34	3.2	1.0	34	3.1	1.1	71	3.1	1.1	30	3.7	0.7	34	3.5	1.0
Activities of daily living	242	3.1	1.1	40	2.8	1.0	34	2.8	1.1	34	3.1	1.2	71	3.1	1.2	30	3.5	1.0	33	3.5	0.9
Relationships	242	3.7	1.0	39	3.9	0.7	34	3.5	0.9	34	3.7	1.1	71	3.5	1.0	30	3.5	1.0	34	3.8	1.1
Living place	243	3.7	1.1	40	3.6	0.8	34	3.1	1.0	34	4.1	1.1	71	3.8	1.2	30	3.6	1.1	34	4.1	1.1
Quality of life	243	3.6	1.0	40	3.5	0.8	34	3.3	0.9	34	4.0	1.0	71	3.3	1.0	30	3.5	0.9	34	3.9	0.9

*Abbreviations*: SWLS: Satisfaction with life scale; LISAT: Life satisfaction questionnaire; PWI: Personal well-being index; WHOQoL: World Health Organization quality of life assessment; AUS: Australia; BRZ: Brazil; CAN: Canada; ISR: Israel; RSA: Republic of South-Africa; USA: United States of America; n: sample size; m: mean raw score; sd: standard deviation of the raw score.

Statistics for the examined measurement properties of the 4 instruments are documented in Table 3. The SWLS showed overall misfit to the Rasch model according to the significant Chi^2 ^test and the PCA eigenvalue. At the item level, 3 out of 5 items showed misfit to the model. In terms of response scale structure, 3 out of 5 items had disordered thresholds. Reliability was high with a value of 0.88.

**Table 3:** Rasch-based fit statistics, ordering of the response scale thresholds, and reliability (n = 243)

Items	δ	SE	z	Chi^2^	df	p	PCA eigen-value	t-test %	τ	r
***SWLS***										
*Overall*				*78.54*	*10*	*0.000**^b^***	*1.97^c^*	*3.3*	*7-steps* *scale*	*0.88*
Ideal life	0.35	0.05	-2.22	13.22	2	0.001***^b^***			disord	
Life conditions	0.15	0.05	-1.70	15.10	2	0.001***^b^***			ord	
Life satisfaction	-0.15	0.05	-1.13	7.21	2	0.027			ord	
Got things I want	-0.25	0.06	0.37	3.10	2	0.212			disord	
Change nothing in life	-0.11	0.05	6.41***^a^***	39.90	2	0.000***^b^***			disord	
										
***LISAT-9***										
*Overall*				*50.60*	*18*	*0.000**^b^***	*2.06^c^*	*7.4**^d^***	*6-step* *scale*	*0.86*
Life as a whole	-0.13	0.07	-2.04	19.15	2	0.000***^b^***			ord	
Self care	0.08	0.05	0.60	1.16	2	0.561			ord	
Vocational situation	0.26	0.06	-0.63	2.65	2	0.266			disord	
Financial situation	0.19	0.06	1.43	0.22	2	0.897			ord	
Leisure situation	0.34	0.06	-0.78	5.45	2	0.066			ord	
Sexual life	0.97	0.06	-0.18	1.42	2	0.491			disord	
Partner relations	-0.30	0.07	2.70***^a^***	11.20	2	0.004***^b^***			disord	
Family life	-0.58	0.06	2.59***^a^***	8.23	2	0.016			disord	
Contact with friends	-0.82	0.07	0.11	1.13	2	0.569			disord	
										
***PWI***										
*Overall*				*15.62*	*16*	*0.480*	*1.96^c^*	*8.7**^d^***	*11-steps* *scale*	*0.92*
Whole life	0.13	0.04	-1.25	5.12	2	0.077			ord	
Living standard	0.02	0.04	1.07	1.56	2	0.457			ord	
Health	0.20	0.04	3.14***^a^***	2.35	2	0.309			ord	
Life achievement	0.05	0.04	1.04	0.62	2	0.735			ord	
Relationships	-0.35	0.04	1.63	1.39	2	0.499			ord	
Feeling safe	-0.11	0.04	-1.44	1.84	2	0.399			ord	
Feel part of community	-0.09	0.04	-0.04	0.39	2	0.824			disord	
Future security	0.14	0.04	-1.11	2.35	2	0.309			ord	
										
***WHOQoL-5***										
*Overall*				*16.43*	*10*	*0.088*	*1.81*	*3.3*	*5-steps* *scale*	*0.78*
Health	0.30	0.09	0.32	1.49	2	0.475			ord	
Activities of daily living	0.68	0.08	-0.29	0.94	2	0.627			ord	
Relationships	-0.37	0.09	1.67	2.04	2	0.361			ord	
Living place	-0.26	0.08	2.05	1.57	2	0.456			ord	
Quality of life	-0.36	0.09	-1.83	10.40	2	0.005***^b^***			ord	

Index:

***a***: Exceeds the critical value of z > +/-2.5

***b***: Below the Bonferroni corrected probability level of p < 0.05/number of items (SWLS and PWI: p < 0.01; LISAT and PWI: p < 0.006)

***c***: Exceeds the decision level for chance distribution of residuals with eigenvalue > 1.9

***d***: Exceeds the 5% boundary for the number of significant independent t-tests based on the PCA results

Abbreviations:

SWLS: Satisfaction with life scale; LISAT: Life satisfaction questionnaire; PWI: Personal well-being index; WHOQoL: World Health Organization quality of life assessment; δ: Item location in logits (delta); SE: Standard error of item location; z: Standard normal distributed test value z; df: Degrees of freedom; p: Probability; PCA: Principal components analysis; t-test %: Percentage of significant independent t-tests; τ: Ordering of the response scale thresholds (tau); r: Person reliability index.

For the LISAT-9, the overall fit statistics (i.e. Chi^2 ^test, PCA eigenvalue, and independent t-test approach) consistently contradict the assumption of unidimensionality. At item level, 3 items out of 9 showed misfit to the Rasch model. In 5 items the response scale thresholds were disordered. The person reliability index was high with a value of 0.86.

For the PWI the Chi^2 ^statistics suggested unidimensionality overall as well as for the individual items. However, the eigenvalue and the t-test approach questioned the assumption of unidimensionality of the instrument. The response scale thresholds were all ordered with the exception of 1 item out of the 8. Reliability was found high with a value of 0.92.

For the WHOQoL-5 all overall statistics confirmed unidimensionality, but one of the items misfitted the model according to the significant Chi^2 ^test result. All response scale thresholds were ordered and reliability was within an acceptable range with a value of 0.78.

The results of the DIF analyses to examine the cross-cultural validity of the 4 instruments are displayed in Table 4. Uniform DIF across countries was found in two items of the SWLS and the WHOQoL-5, three items of the LISAT-9 and four items of the PWI. Non-uniform DIF was found only in the item "Leisure situation" of the LISAT-9 (data not shown). For the SWLS and the LISAT-9 the data from Israel showed most frequently significant differences from the other countries. For the PWI, the data from Australia and Canada showed most frequently significant differences to other countries. For the WHOQoL-5 this was the case for the data from Canada (data for post-hoc tests not shown).

**Table 4:** DIF across countries prior to and after applying the post-hoc strategies (n = 243)

Items	Prior modification				After modification			
		
	MS	F	df	p	MS	F	df	p
***SWLS***					***Strategy 2***			
Ideal life	1.72	3.36	5	0.006	0.86	1.47	5	0.202
Life conditions	1.54	2.74	5	0.020	0.58	0.88	5	0.494
Life satisfaction	1.64	2.70	5	0.022	1.55	2.26	5	0.050
Got things I want	3.03	4.01	5	0.002***^b^***	deleted			
Change nothing in life	15.15	11.20	5	0.000***^b^***	deleted			
								
***LISAT-9***					***Strategy 4***			
Life as a whole	1.45	2.35	5	0.042	0.76	1.15	5	0.333
Self care	3.23	3.64	5	0.004***^b^***	2.43	2.85	5	0.016
Vocational situation	1.34	1.67	5	0.142	0.97	1.37	5	0.237
Financial situation	7.13	8.08	5	0.000***^b^***	merged			
Leisure situation	4.17	6.64	5	0.000***^b^***	merged			
Sexual life	0.87	1.04	5	0.3959	2.01	1.90	5	0.095
Partner relations	2.27	1.65	5	0.1513	deleted			
Family life	3.74	3.38	5	0.0058	deleted			
Contact with friends	0.72	0.80	5	0.5485	2.21	2.05	5	0.073
*Merged item 4 and 5*					*1.19*	*1.90*	*5*	*0.095*
								
***PWI***					***Strategy 3***			
Whole life	1.80	2.59	5	0.027	1.783	2.53	5	0.030
Living standard	2.08	2.28	5	0.048	2.094	2.25	5	0.051
Health	9.22	9.59	5	0.000***^b^***	merged			
Life achievement	1.72	1.80	5	0.114	1.792	1.84	5	0.106
Relationships	4.44	4.65	5	0.001***^b^***	merged			
Feeling safe	3.80	5.63	5	0.000***^b^***	merged			
Feel part of community	1.82	2.20	5	0.055	1.811	2.16	5	0.060
Future security	2.77	3.88	5	0.002***^b^***	merged			
*Merged item 3 and 8*					*6.683*	*9.05*	*5*	*0.000**^b^***
*Merged item 5 and 6*					*0.286*	*0.39*	*5*	*0.858*
								
***WHOQoL-5***					***Strategy 3***			
Health	2.90	3.85	5	0.002***^b^***	merged			
Activities of daily living	1.50	2.04	5	0.075	1.41	2.04	5	0.074
Relationships	2.32	2.50	5	0.031	2.14	2.46	5	0.034
Living place	2.97	2.99	5	0.012	2.69	2.89	5	0.015
Quality of life	2.36	4.13	5	0.001***^b^***	merged			
*Merged item 1 and 5*					*0.80*	*1.47*	*5*	*0.200*

Index:

***b***: Below the Bonferroni corrected probability level of p < 0.05/number of items (SWLS and PWI: p < 0.01; LISAT and PWI: p < 0.006)

Abbreviations:

SWLS: Satisfaction with life scale; LISAT: Life satisfaction questionnaire; PWI: Personal well-being index; WHOQoL: World Health Organization quality of life assessment; DIF: Differential item functioning; MS: Mean square sum of residuals; F: F-distributed test value; df: Degrees of freedom; p: probability

Table 5 shows the statistics about instrument and item fit, response scale structure, and reliability for the 4 different strategies applied to enhance the measurement properties and the cross-cultural validity of the 4 instruments. Also, Table 4 contains the results of the final check for DIF after having identified the optimal option for handling the data.

**Table 5:** Rasch-based statistics for the different strategies applied to enhance the metric properties of the instruments (n = 243)

Items	Strategy 1: Collapsing response scale						Strategy 2: Deleting misfitting items						Strategy 3: Merging DIF items						Strategy 4: Merging DIF items (Option 1 or 2)					
				
	δ	SE	τ	z	p	r	δ	SE	τ	z	p	r	δ	SE	τ	z	p	r	δ	SE	τ	z	p	r
**SWLS**	recoding: 0 1 1 2 2 3 4																		no DIF in selected option					
*Overall*					0.990	0.87					*0.250*	*0.90*					0.000***^b^***	0.89						
Ideal life	0.48	0.11	ord	-0.83	0.811		*0.39*	*0.07*	*disord*	*-0.88*	*0.119*		0.32	0.06	ord	-1.11	0.014							
Life conditions	0.077	0.11	ord	0.08	0.910		*0.06*	*0.07*	*ord*	*-0.43*	*0.220*		0.11	0.06	ord	-0.56	0.006***^b^***							
Life satisfaction	-0.56	0.11	ord	0.75	0.870		*-0.45*	*0.07*	*ord*	*0.43*	*0.758*		-0.24	0.06	ord	0.07	0.165							
Got things I want	del						*del*						merg											
Change nothing in life	del						*del*						merg											
Merged item 4. and 5.													-0.19	0.04	ord	2.67***^a^***	0.038							
																								
**LiSAT**	recoding: 0 1 1 1 2 3																		*recoding: 0 1 1 1 2 3*					
*Overall*					0.074	0.81					0.106	0.80					0.000***^b^***	0.86					*0.125*	*0.81*
Life as a whole	-0.44	0.12	ord	-1.72	0.022		del						-0.08	0.07	ord	-1.96	0.000***^b^***		*-0.35*	*0.122*	*ord*	*-1.38*	*0.037*	
Self care	-0.08	0.10	ord	0.32	0.739		-0.08	0.06	ord	1.69	0.558		0.12	0.05	ord	0.68	0.626		*0.00*	*0.098*	*ord*	*0.65*	*0.671*	
Vocational situation	0.27	0.10	ord	-1.82	0.382		0.10	0.06	disord	0.25	0.125		0.30	0.06	disord	-0.55	0.321		*0.34*	*0.105*	*ord*	*-1.35*	*0.454*	
Financial situation	0.12	0.11	ord	0.53	0.305		del						merg						*merg*					
Leisure situation	0.41	0.11	ord	-1.55	0.279		0.18	0.06	ord	-1.22	0.014		merg						*merg*					
Sexual life	1.39	0.11	ord	1.93	0.055		0.86	0.06	disord	0.50	0.777		0.995	0.06	disord	-0.11	0.476		*1.45*	*0.112*	*ord*	*2.23*	*0.049*	
Partner relations	del						del						-0.27	0.07	disord	2.77***^a^***	0.008		*del*					
Family life	del						del						-0.53	0.06	disord	2.74***^a^***	0.021		*del*					
Contact with friends	-1.67	0.11	ord	1.97	0.517		-1.06	0.07	disord	0.58	0.481		-0.77	0.07	disord	0.15	0.562		*-1.63*	*0.110*	*ord*	*2.37*	*0.427*	
Merged item 4. and 5.													0.24	0.04	ord	-1.66	0.279		*0.18*	*0.078*	*disord*	*-2.25*	*0.616*	
																								
**PWI**	no recoding (one item disordered)																							
*Overall*											0.346	0.92					*0.710*	*0.92*						
Whole life							0.17	0.04	ord	0.26	0.250		*0.11*	*0.04*	*ord*	*-0.70*	*0.121*							
Living standard							0.06	0.04	ord	0.97	0.050		*0.00*	*0.04*	*ord*	*1.66*	*0.427*							
Health							del						*merg*											
Life achievement							0.09	0.04	ord	1.28	0.857		*0.03*	*0.04*	*ord*	*1.62*	*0.646*							
Relationships							-0.35	0.04	ord	1.68	0.207		*merg*											
Feeling safe							-0.08	0.04	ord	-1.04	0.481		*merg*											
Feel part of community							-0.06	0.04	disord	0.06	0.598		*-0.09*	*0.041*	*disord*	*0.495*	*0.730*							
Future security							0.18	0.04	ord	-0.46	0.681		*merg*											
Merged item 3. and 8.													*0.19*	*0.03*	*ord*	*0.54*	*0.949*							
Merged item 5. and 6.													*-0.23*	*0.03*	*ord*	*-1.00*	*0.501*							
																								
**WHOQOL**	no recoding (no disorder)																							
*Overall*											0.340	0.81					*0.567*	*0.76*						
Health							0.12	0.10	ord	0.15	0.371		*merg*											
Activities of daily living							0.68	0.09	ord	-0.30	0.240		*0.58*	*0.08*	*ord*	*-0.29*	*0.604*							
Relationships							del						*-0.41*	*0.08*	*ord*	*1.59*	*0.582*							
Living place							del						*-0.34*	*0.08*	*ord*	*1.92*	*0.475*							
Quality of life							-0.80	0.10	ord	0.07	0.376		*merg*											
Merged item 1. and 5.													*0.16*	*0.06*	*ord*	*-2.20*	*0.208*							

Index:

***a***: Exceeds the critical value of z > +/-2.5

***b***: Below the Bonferroni corrected probability level of p < 0.05/number of items (SWLS and PWI: p < 0.01; LISAT and PWI: p < 0.006)

Abbreviations:

SWLS: Satisfaction with life scale; LISAT: Life satisfaction questionnaire; PWI: Personal well-being index; WHOQoL: World Health Organization quality of life assessment; DIF: Differential item functioning; δ: Item location in logits (delta); SE: Standard error of item location; τ: Ordering of the response scale thresholds (tau); z: Standard normal distributed test value z; p: Probability of the Chi^2 ^test; r: Person reliability index; del: Item deleted due to misfit; merg: Item merged due to DIF; ord: Response scale thresholds ordered; disord: Response scale thresholds disordered

Strategy 2 was regarded as the optimum choice for the SWLS. Two misfitting items were deleted using the step-wise data purification procedure. With this handling of the data, item fit and response scale order were achieved, and no DIF was apparent.

Strategy 4 was regarded the optimum choice for handling the data for the LISAT-9. Only after collapsing the response options, deleting two misfitting items and merging another two items with DIF were all the remaining items fitting, the response scale thresholds ordered (with one exception), and DIF not present.

Strategy 3 appeared the optimum choice for the PWI. The scores of the four items that displayed DIF prior to applying any post-hoc strategies were merged into two items, which lead to no item misfit and no response scale disorder. However, one of the merged items remained inconsistent across countries and displayed DIF.

Strategy 3 was also the optimum choice for the WHOQoL-5. After merging the scores of those two items which initially indicated DIF, all items fitted the Rasch model, the response scale thresholds were ordered, and no DIF was found.

## Discussion

The study examined the metric properties of the Satisfaction with Life Scale (SWLS), the Life Satisfaction Questionnaire (LISAT), the Personal Well-Being Index (PWI) and the 5-item World Health Organization Quality of Life Assessment (WHOQoL-5) in a cross-country sample of persons with SCI based on Rasch analysis. Although all instruments displayed metric problems in the analyses and showed cross-country bias at first, it was possible to identify post-hoc strategies to ameliorate those problems. Such strategies can also be used in further studies to enhance the metric comparability of data across countries. The two instruments which performed best overall in this comparison in terms of reliability, dimensionality, response scale structure, and cross-cultural validity were the WHOQoL-5 and the PWI, prior as well as after applying the post-hoc strategies.

### Reliability

In the current study, high values of the person reliability index were found for all four instruments. The person reliability index was similar for the WHOQoL-5 and for the SWLS in our study to alpha coefficients reported in the literature in different samples and countries, including also persons with spinal cord injuries [40,41,43,63,65-67]. However, for the PWI and the LISAT-9, the reliability index was higher than reliability measures reported earlier [37,53,54,61,62]. The person reliability index is the Rasch-based counterpart of Cronbach's alpha. In this study, alpha coefficients could not be calculated because of missing data. Rasch analysis, however, not only deals readily with missing data [84], but in general the person reliability index can also have the advantage of being a more conservative estimate of reliability under certain circumstances, e.g. when alpha may be inflated due to the number of items or the sample variance [85].

### Dimensionality

In line with an earlier study using structural equation modeling [67], unidimensionality can be assumed for the WHOQoL-5. For the PWI, previous studies indicated unidimensionality, which is partially supported by the statistics in this analysis [60,62]. Although unlike previous authors, we included the first overall item in the analyses [37], in the item-wise examination, this overall item fitted the model along with the domain-specific items.

The assumption of unidimensionality was rejected for the LISAT-9 and the SWLS. Earlier studies, as well as the findings presented here, suggest that more than one dimension is assessed by the LISAT [23,53]. In this study, with deleting the two items "partner relations" and "family life" unidimensionality of the remaining items was established. The item "partner relations" had far more missing data than any of the other items (see Table 2), which might have caused metric irregularities. However, the standard error of the estimates was not larger compared to the other items, indicating acceptable precision of estimation. However, a potential explanation how these two items differ from the others could lay in the specific meaning of the items in the context of SCI and in the specific experiences of the affected persons. While the other items may be related to the experienced difficulties and problems in body functions, activities and participation imposed by SCI (e.g. difficulties in sexuality, less contact with friends), the partner and family life items may be related to the more stable, positive, and support providing relationships [86]. Thus, the difference between the separate dimensions identified in the statistical analyses might be interpreted conceptually as negative versus positive experience, problems in own functioning versus support by others.

The results regarding the unidimensionality of the SWLS contradict the findings of several earlier studies, which demonstrated a single underlying dimension [21,40-43,47,50]. In this study the last two items ("If I could live my life over, I would change almost nothing" and "So far I have gotten the important things I want in life") had to be removed before unidimensionality was achieved for the remaining three. A study from France using structural equation modeling found no support for the unidimensionality of the SWLS in a general population sample and the authors proposed to take the last two items separately [87]. They suggest that the semantic structure of those two items, which relate to the past, may explain the inconsistency among the items. In the current study the sample consisted of persons who have met with a major life event in the past, namely SCI. One thing that persons with SCI might want to change in the past and might be strongly dissatisfied with is the SCI itself [47]. In the context of SCI, it could be hypothesized that the first items (related to present life satisfaction) of the SWLS might be connected to acceptance, the last two items to grief and regret. These different connotations might explain in line with the suggestion of Vautier et al. (2004) the observed inconsistency and disjunction among the items within the instrument.

### Response scale structure

Considering the response scale structure of the instruments, the results suggest that the 5-steps scale of the WHOQOL-5 ("very dissatisfied", "dissatisfied" "neither satisfied nor dissatisfied", "satisfied", "very satisfied") and the 11-steps numeric rating scale of the PWI ("completely dissatisfied" to "neutral" to "completely satisfied") have the expected ordering and persons with SCI could differentiate between the steps consistently when responding to the items.

For the SWLS and the LISAT the response scale structure showed disorder in several items. For the SWLS, after removing the last two items for misfit, only one disordered item ("ideal life") remained. For the LISAT-9 the original 6-step rating scale was reduced to a 4-step solution in this study. The optimal solution in the post-hoc analyses appeared to be the merging of the response options "dissatisfying", "rather dissatisfying" and "rather satisfying". This merging of the response options parallels the cut-off used by Fugl-Meyer to dichotomize item scores (1-4 = satisfied/5-6 = unsatisfied) placing the "rather satisfying" option in the unsatisfied category [53]. Accordingly, future studies could test the metric properties and usefulness of a modified 4-step scale for the LISAT with a suggested structure as "very dissatisfying", "dissatisfying", "satisfying", and "very satisfying".

### Cross-cultural validity

The current findings hint at potential cross-country bias in all four examined instruments largely in line with existing research. In the case of the SWLS, two earlier studies using different methodologies found indications that the comparability and interpretability of the scores across countries is not consistent [51,52], which is now supported in an SCI sample.

Lau et al (2005) found cross-cultural differences in the performance of the PWI between an Australian and a Hong Kong Chinese population and suggested that cultural response bias would be a plausible explanation for the differences [61]. Our results in SCI showed DIF for 4 of the PWI items across the 6 countries, and Australia was among the countries which showed strongest deviation from the other five (beside Canada). However, by merging the scores of those items which had DIF, the deviations proved to be balanced out. Thus, at the level of the summary score, cross-country comparability may be possible.

Schmidt et al (2006) examined DIF for the Eurohis-QoL-8 instrument, which is a selection of 8 items out of the WHOQOL-BREF and which includes the 5 items used in this study [67]. They found acceptable cross-cultural properties in their instrument which is in line with the findings here for the reduced 5-item version. Again, the minor deviation in the first DIF analyses could be alleviated by merging the two items "health" and "quality of life" to establish cross-country comparability of the summary score.

Although the LISAT has been used in cross-country studies [25,26], those did not examine potential bias between the different language versions of the instrument. In this study, the post-hoc analyses showed that acceptable metric properties could only be achieved for the LISAT by applying the whole range of modification strategies, including the collapsing of response options, the deletion of items and the merging of item scores.

### Limitations

The study is subject to several methodological limitations. The major drawback of the study is the low sample size in the individual countries. For this reason certain statistical techniques for assessing psychometric characteristics and handling DIF could not be applied, e.g. the item-splitting method suggested by Tennant et al [33]. However, the overall sample size was sufficient to reliably sustain the performed analyses [88]. According to Linacre (1994) a sample size of n = 250 is sufficient to achieve stable item parameters. In the current analyses the stability of the parameters was high, obvious from the small standard errors of the item parameters (SE = 0.04-0.09, see Table 3). Secondly, as the study included a convenience sample of persons with SCI, selection bias cannot be ruled out and the generalizability of the results may be compromised. Third, the quality of the Portuguese and Hebrew language versions of the questionnaires were not tested prior to their use in these data collections. Fourth, as a more current development, the PWI includes a further item on spirituality, which was not yet taken up in the data collections for this study. Fifth, in these analyses, only basic psychometric characteristics (i.e. reliability, unidimensionality) were considered, but features like stability or sensitivity to change were not examined. Sixth, the DIF analyses only focused on potential cross-country biases, but were not extended to other factors that might influence the participants' responses, e.g. sociodemographic factors or depression. Finally, the post-hoc solutions shown in this study can be considered "optimum" only in the current sample, and in other studies the results may look different. However, we have shown that using these strategies data can be handled in a way that increases the confidence in the metric quality and interpretability of the data.

## Conclusions

The Rasch analyses of the four quality of life instruments showed that the raw scores were not consistently comparable across countries at first in an international SCI sample. However, by accounting for DIF across countries in a way that the requirements of the Rasch model are met, the scores can become comparable. Following the post-hoc procedures the items of the WHOQOL-5 and the PWI worked in a consistent and expected way in all countries. Thus, the differences between countries assessed by these instruments could potentially show cross-culturally valid differences in the responses of the persons. In contrast, summary scores of the LISAT-9 and the SWLS have to be interpreted with caution. The findings of the current study can be especially helpful to select instruments for international research projects in spinal cord injury.

## Competing interests

The authors declare that they have no competing interests.

## Authors' contributions

SG contributed to the conception and design of the study, the conception and interpretation of the statistical analyses, and drafted the manuscript. BF conducted the statistical analyses, contributed to the interpretation of data, the drafting and revision of the manuscript. IK contributed to the acquisition and management of the data and revised the manuscript. MP contributed to the conception and design of the study, the acquisition of data, the interpretation of the statistical analyses, and revised the manuscript. All authors read and approved the final manuscript.
